# A recursive partitioning approach to investigating correlates of self-rated health: The CARDIA Study

**DOI:** 10.1016/j.ssmph.2017.12.002

**Published:** 2017-12-15

**Authors:** Shilpa Nayak, Alan Hubbard, Stephen Sidney, S. Leonard Syme

**Affiliations:** a Department of Public Health and Policy, The Whelan Building, Quadrangle, The University of Liverpool, Liverpool L69 3GB, UK; b School of Public Health, The University of California, Berkeley, CA 94720, USA; c Kaiser Permanente Northern California Division of Research, 2000 Broadway, Oakland, CA 94612, USA

**Keywords:** Self-rated health, Health determinants, Recursive partitioning methods, Classification tree analysis, Random forests

## Abstract

Self-rated health (SRH) is an independent predictor of mortality; studies have investigated correlates of SRH to explain this predictive capability. However, the interplay of a broad array of factors that influence health status may not be adequately captured with parametric multivariate regression. This study investigated associations between several health determinants and SRH using recursive partitioning methods. This non-parametric analytic approach aimed to reflect the social-ecological model of health, emphasizing relationships between multiple health determinants, including biological, behavioral, and from social/physical environments. The study sample of 3648 men and women was drawn from the year 15 (2000–2001) data collection of the CARDIA Study, USA, in order to study a young adult sample. Classification tree analysis identified 15 distinct, mutually exclusive, subgroups (eight with a larger proportion of individuals with higher SRH, and seven with a larger proportion of lower SRH), and multi-domain risk and protective factors associated with subgroup membership. Health determinant profiles were not uniform between subgroups, even for those with similar health status. The subgroup with the largest proportion of higher SRH was characterized by several protective factors, whilst that with the largest proportion of lower SRH, with several negative risk factors; certain factors were associated with both higher and lower SRH subgroups. In the full sample, physical activity, education and income were highest ranked by variable importance (random forests analysis) in association with SRH. This exploratory study demonstrates the utility of recursive partitioning methods in studying the joint impact of multiple health determinants. The findings indicate that factors do not affect SRH in the same way across the whole sample. Multiple factors from different domains, and with varying relative importance, are associated with SRH in different subgroups. This has implications for developing and prioritizing appropriate interventions to target conditions and factors that improve self-rated health status.

## Highlights

• Self-rated health (SRH) is an independent predictor of morbidity and mortality.
• Partitioning identified 15 distinct subgroups by SRH status and multidomain factors.
• Differing risk factor profiles have implications for strategies to improve SRH.
• Physical activity, education, income ranked highest overall in relation to SRH.
• This approach reflects a social-ecological perspective on correlates of SRH.

## Introduction

Self-rated health (SRH) is recognized as a valid assessment of health status, and independent predictor of mortality (Idler & Benyamini, 1997). Correlates of SRH have been investigated with a view to explaining this predictive capacity, identifying independent determinants of SRH from demographic, lifestyle, medical, and psychosocial domains. Lower health ratings have been associated with increasing age (Daniilidou et al., 2004, McFadden et al., 2008, Pleis et al., 2010, Shadbolt, 1997), being female (Daniilidou et al., 2004, Eriksson et al., 2001, Franks et al., 2003), and being of black (Franks et al., 2003) or Hispanic ethnicity (Franks et al., 2003, Pleis et al., 2010) compared with white. Higher education and income are positively associated with higher SRH status (Bobak et al., 1998, Franks et al., 2003, Molarius et al., 2007, Pleis et al., 2010, Shields and Shooshtari, 2001). Behavioral factors associated with poorer SRH include diet, physical inactivity, smoking, alcohol consumption, and higher body weight (Benyamini and Leventhal, 1999, Ferraro and Yu, 1995, Manderbacka et al., 1999, Molarius et al., 2007). Associations with SRH have also been observed for chronic medical morbidity and physical functioning, fatigue, lack of energy, number of medications, and negative affect (Benyamini and Leventhal, 1999, Kempen et al., 1998). Psychosocial variables related to low SRH include lack of social support, sense of community belonging (Shields, 2008), low perceived control over life, indicators of happiness, and working conditions (Benyamini and Leventhal, 1999, Bobak et al., 1998, Molarius et al., 2007). Cross-sectional studies have demonstrated higher rates of poor perceived health in people who also report higher levels of social isolation, negative life events, depression, job problems, unhappiness, life dissatisfaction and unemployment. Poor SRH may be a common feature linking psychosocial factors to disease outcomes via a decrease in host resistance (G. A. Kaplan & Camacho, 1983; Syme & Berkman, 1976).

Considering a number of studies have sought to explore the determinants or correlates of SRH, there are two issues to consider – the lack of consensus across studies regarding the particular factors SRH represents, and the methods used. First, the variations in health determinants are unsurprising when considering dissimilar samples or populations, based on age, (Giron, 2012, McFadden et al., 2008, Tremblay et al., 2003, Verropoulou, 2009) occupation, (Haddock et al., 2006, Mikolajczyk et al., 2008, Singh-Manoux et al., 2006, Vaez and Laflamme, 2002, Vingilis et al., 1998) and geography (Ahmad et al., 2005, Asfar et al., 2007, Cott et al., 1999, Daniilidou et al., 2004, Darviri et al., 2012, Franks et al., 2003, Giron, 2012, Shadbolt, 1997, Sun et al., 2007, Tremblay et al., 2003, Xu et al., 2010). In fact, when attempting to unpack the concept of SRH in a particular population, the value is in capturing the unique determinants of health that are most important in that context and group. Second, many previous quantitative studies have used parametric multivariate regression. When considering the influence of the broad spectrum of determinants which may influence SRH, it may be difficult to satisfy the requirements of these models, in terms of underlying data structure of predictors, and to examine a large number of variables and interactions. A review of a sample of fifty-six published studies on determinants of SRH identified several problems related to multivariate regression modeling, including over-fitting, nonconformity to a linear gradient, and lack of reporting of tests for interactions; though SRH is a multifaceted measure, most studies did not cover its various components concomitantly (Mantzavinis, Pappas, Dimoliatis, & Ioannidis, 2005).

Knowledge of the relationship between single predictors and outcomes is clearly essential. However, the strength of conceptualising the potential determinants of SRH, or other health outcomes, using CTA is that it builds upon individual factor-outcome relationships, typically gained from parametric regression models, and adds detail on interactions between influences from multiple domains. This may better reflect how multiple influences on health interact in reality; particularly also where relationships between health determinants are not necessarily simple, or represented by linear models.

For some diseases, even well studied biological risk factors alone fail to account for all the disease that occurs, whilst psychosocial factors and socioeconomic conditions are linked with multiple conditions (Syme, 2004). Single elements of the broad range of health determinants reflect only some aspect of health but without consideration of cofactors, may be incomplete predictors of overall health status (Portrait, Lindeboom & Deeg, 1999). This study approach is based on the social-ecological model of health, which emphasizes relationships between multiple health determinants, from domains including biology, behavior and the social and physical environments, and assumes that health is affected by their interaction (Dahlgren G, 1991, Gebbie et al., 2003). Accordingly, ecological research seeks to include as many theoretically relevant ecological contrasts as possible, in contrast to classical experiments focusing on a single variable, and attempting to control out potential confounders (Bronfenbrenner, 1977). Recursive partitioning methods can identify the wide range of interacting influences on individuals that confer susceptibility to illness, or support resilience and wellbeing, and their relative importance to the outcome; this can inform public health action aimed at improving harmful conditions and promoting protective factors that improve health status.

The aim of this study is to demonstrate the use of recursive partitioning methods (classification tree analysis, and random forests) for investigating multi-domain correlates of SRH status. We show that these methods offer valuable insight, which is distinct to that gained by parametric regression models, on the joint impact of multiple factors, and the way in which varying combinations of health determinants influence SRH in different subgroups. Classification tree analysis (CTA) is useful in a public health context as it segments the study sample into mutually exclusive population subgroups with selected common characteristics in relation to SRH status (Forthofer & Bryant, 2000), and identifies the risk and protective factors associated with subgroup membership (Breiman, Friedman, Olshen, & Stone, 1984).

## Methods

### Data: The CARDIA Study

Cross-sectional data used for the analysis were collected during the CARDIA Study (Coronary Artery Risk Development in Young Adults). The CARDIA cohort study began in 1985 with 5115 black and white men and women, aged between 18 and 30 years (1.1% of participants were 17–35 years), recruited in Birmingham, Alabama; Chicago, Illinois; Minneapolis, Minnesota; and Oakland, California, USA. At baseline, 54.5% were women (n=2787), 45.5% were men (n=2328); 48.4% were white (n=2478), and 51.6% were black (n=2637). For the current study, data were taken from the year 15 examination of the CARDIA cohort, as the focus was on young adults, conducted in 2000–2001, through interviewer and self-administered questionnaires (with the exception of race/ethnicity information taken from the 1985–1986 data collection, and family history taken from the 1995 data collection). From 5115 participants, 3672 were followed up in year 15 (72% of the original cohort at baseline). From the year 15 group, all remaining participants who had a response for SRH, and were coded as male or female, were included in the final study sample of 3648 participants (one participant withdrew from the study in year 25, and is excluded from the analysis of year 15 data).

### Study variables

#### Outcome variable

SRH was assessed on a five-point scale, by the question, *“In general would you say your health is excellent, very good, good, fair or poor?”* Responses were categorized by grouping together excellent or very good as ‘higher’ SRH, and responses of good, fair or poor, as ‘lower’ SRH. Responses of very good or excellent were grouped as higher SRH, as they were more definite positive statements of better health; respondents may have regarded a response of good, being the center of a 5-point scale, as a neutral or ‘average’ value. This grouping also resulted in more equal group sizes.

#### Predictor variables

A broad range of health determinants were included as predictor variables, representing age, sex and hereditary factors; individual lifestyle factors and medical history; social and community influences; living and working conditions (Appendix Table A1).

### Recursive partitioning

**Fig. 1:**
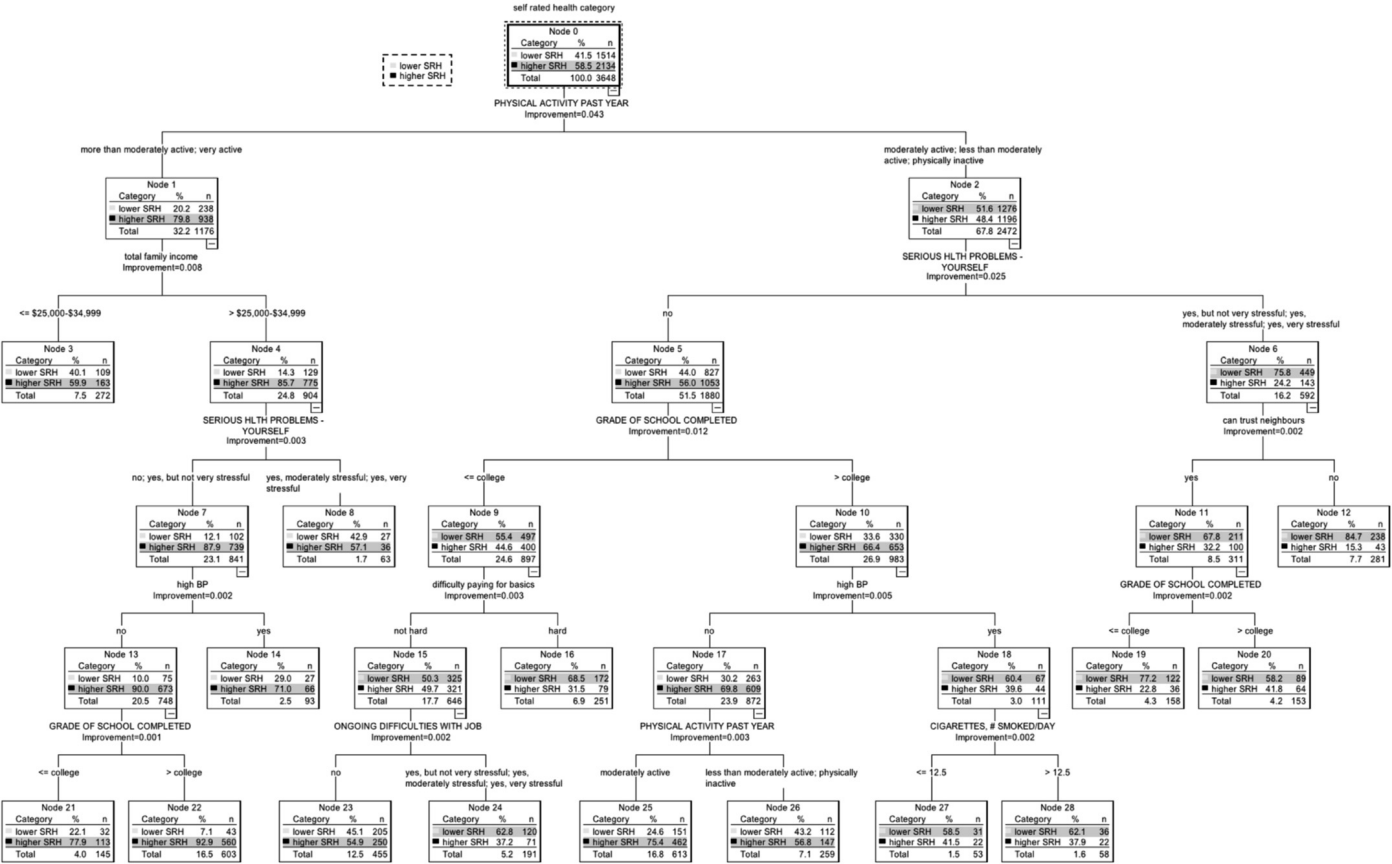
Classification Tree Analysis of Self-Rated Health Status and Health Determinants, the CARDIA Study Year 15, USA. There are 15 mutually exclusive subgroups (terminal nodes) in the tree model. However, all subgroups produced during the construction of the tree model are numbered (1–28), and so the text refers to subgroup numbers higher than 15.

CTA constructs a single tree model. The entire data sample (the root node) is first partitioned into 2 subgroups (child nodes), based on a binary question relating to a predictor variable (e.g., is income <=$25,000-$34,999?). The proportion of cases in the node answering, e.g., ‘yes’, goes to one child node, and proportion answering ‘no’, to the other child node. At every node, partition of the sample is based on that predictor variable which maximizes the goodness-of-split function, i.e. splitting creates nodes or subgroups that are more homogenous or ‘purer’ than the data in the original parent grouping (Breiman et al., 1984). Each subsequent node that is split is referred to as a parent node; and one that is not split further is a terminal node. This process of splitting the study sample is repeated multiple times until a predetermined number of individuals exist in each subgroup, or the largest possible tree is grown. The subgroups formed, and the splitting predictor variables, are shown in the form of a tree diagram (Fig. 1). A process of ‘pruning’ refines the tree model: potentially unnecessary nodes and branches are removed to create a sequence of smaller trees. Cross-validated risk is used to choose the tuning parameters of the final optimal tree, which include, for instance, the number of subgroups. This avoids overfitting that can result in splits that add nothing (or detract) from the predictive precision of the tree. Cross-validation gives an internal estimate of misclassification by the tree model (Breiman et al., 1984). Ten-fold cross-validation is a commonly used value. In this procedure, the sample is split into ten equal subsamples; each subsample in turn is withheld whilst the remaining nine are used to build a test tree. The remaining subsample is used as an independent test sample. The 10-fold cross-validation error estimate is calculated by averaging across all 10 trees. In the final tree model, the terminal nodes represent the entire data sample of individuals split into mutually exclusive subgroups.

Random forests analysis builds on the single tree produced by CTA with an ensemble (or ‘forest’) of classification trees, improving accuracy and producing a more robust importance ranking of the predictor variables associated with SRH (Breiman, 2001a). Random Forests are constructed by drawing a bootstrap sample (where n observations are sampled with replacement from the original sample), to which recursive partitioning is applied (Breiman, 2001a, Zhang and Singer, 2010). At each node, from the original complete set of predictor variables, a random subset is selected, and the tree splits are restricted based on these, dividing the study sample; this reduces correlation between trees. Trees are generated without pruning. A bagging (bootstrap aggregation) process decreases the variance created by the lack of pruning (Goldstein, Hubbard, Cutler, & Barcellos, 2010): in this, each tree created in the ensemble is produced using a different bootstrap sample from the study dataset, whilst approximately one third of cases are unselected. These form the ‘out-of-bag’ sample, which is put into the tree to get a classification. Splitting of the data continues until the node is homogenous (for SRH status), or there are no more predictor variables on which to split. These steps are repeated a predetermined number of times to form a forest of trees. The out-of-bag error rate is an integral internal error rate produced as a result of the bagging process (Goldstein et al., 2010, Strobl et al., 2008, Strobl et al., 2009).

### Statistical analyses

Descriptive summaries were produced for the study sample. The chi-square test for independence (with Yates’ continuity correction for 2×2 tables) was used to assess the bivariate relationship between individual categorical predictor variables and SRH, and Mann Whitney U Tests for continuous predictor variables and SRH, following tests for normality of distribution. Responses of ‘don’t know’ to a question were grouped with, and treated as, missing data. Parameters for the classification tree model were specified as: cross validation with 10 sample folds; minimum number of cases - 100 for parent node and 50 for child node (the minimum size of subgroups created by the tree). Characteristics of the subgroups in the tree model with the highest and lowest proportion of good SRH were compared with the remaining sample using z-tests. Data analyses were carried out using IBM SPSS Statistics v21.

Random forests analysis was applied using the Random Forests package in R, through the R integration package RanFor (R version 2.14.0 Copyright © 2011 The R Foundation for Statistical Computing [http://www.r-project.org]) in IBM SPSS Statistics v21. One thousand trees were specified in the random forests model to generate variable importance measures, and the default value was used for the number of predictor variables sampled at each node; for classification trees, this is the square root of the number of predictors. The parameters were set to impute missing values for scale variables as the variable’s median value, and for categorical variables, as the modal value (Liaw & Wiener, 2002). Variable importance ranking was based on the Gini Index as a measure of node impurity; as the study sample is split in the analysis, the resulting subgroups are more homogenous or pure than data in the initial group (or ‘parent’ node); each split is based on the predictor variable, and its split point, that most reduces the impurity of the initial group or parent node.

## Results

In the study sample (n=3648), the distribution of SRH status was excellent, 17% (n=631); very good, 41% (1503); good, 32% (n=1166); fair, 9% (n=316), and poor, 1% (n=32): This resulted in 58.5% of individuals (n=2134) being grouped in the ‘higher’ SRH category, and 41.5% (n=1514) in the ‘lower’ SRH category. The mean age was 40.2 years; 55.8% were women (n=2036) and 44.2% were men (n=1612); 52.6%, (n=1920) individuals were white, and 47.1% (n=1717) were black (0.3%, n=11 were Hispanic. CARDIA was designed to be a cohort studying black and white participants. However, information on ethnicity was collected and 11 participants were classified in the study as Hispanic).

Continuous predictors had a non-normal distribution. There were bivariate significant associations between SRH status and sex, race/ethnicity, physical activity rating, cigarette smoking, perceived social support and neighborhood cohesion, total family income, home ownership, unemployment, health insurance, difficulty paying for basics, optimism for the future, control over life events, and chronic burden due to serious on-going personal health problem, at significance level p<0.05 (Appendix Table A2). In the lower SRH category, there were significantly higher values for number of fast food meals per week, cigarettes smoked per day, and liquor drinks per week (p<0.05). The number of wine drinks per week was higher in the higher SRH category (p<0.05).

### Classification tree analysis

Fifteen mutually exclusive subgroups (terminal nodes) were formed in the classification tree model which had an overall misclassification rate of 31% based on cross-validation (Fig. 1). Summary characteristics of the 15 subgroups are described in Table 1. There were 8 subgroups in the study sample with predominantly higher SRH, ranging in proportion from 54.9% to 92.9% of the subgroup. There were 7 subgroups with predominantly lower SRH, ranging from 58.2% to 84.7% of the subgroup. The primary split of the study sample in the tree was on physical activity rating, with a higher level of physical activity associated with higher SRH.

**Table 1:** Subgroups by classification tree analysis (Fig. 1) of self-rated health status and health determinants, the CARDIA Study, Year 15, USA.

**Subgroup/ Node number (predominant SRH status)**	**Number in subgroup N**	**% ‘higher’ self-rated health**	**Description of node characteristics**
22	603	92.9	Physical activity in the past year is more than moderately active or very active; total family income is >=$25,000-$34,999; chronic burden due to serious on-going personal health problem (no, or yes, but not very stressful); no history of high blood pressure; grade of school completed is higher than college
higher			
			
21	145	77.9	Physical activity in the past year is more than moderately active or very active; total family income is >=$25,000-$34,999; chronic burden due to serious on-going personal health problem (no, or yes, but not very stressful); no history of high blood pressure; grade of school completed is less than college
higher			
			
25	613	75.4	Physical activity in the past year is moderately active or less than moderately active, or physically inactive; no chronic burden due to serious ongoing personal health problem; grade of school completed is higher than college; no history of high blood pressure; physical activity in the past year is moderately active
higher			
			
14	93	71.0	Physical activity in the past year is more than moderately active or very active; total family income is >=$25,000-$34,999; chronic burden due to serious on-going personal health problem (no, or yes, but not very stressful); history of high blood pressure
higher			
			
3	272	59.9	Physical activity in the past year is more than moderately active or very active; total family income is <=$25,000-$34,999
higher			
			
8	63	57.1	Physical activity in the past year is more than moderately active or very active; total family income is >=$25,000-$34,999; chronic burden due to serious on-going personal health problem (yes, moderately stressful or yes, very stressful)
higher			
			
26	259	56.8	Physical activity in the past year is moderately active or less than moderately active, or physically inactive; no chronic burden due to serious ongoing personal health problem; grade of school completed is higher than college; no history of high blood pressure; physical activity in the past year is less than moderately active or physically inactive
higher			
			
23	455	54.9	Physical activity in the past year is moderately active or less than moderately active, or physically inactive; no chronic burden due to serious ongoing personal health problem; grade of school completed is less than college; degree of difficulty paying for basics is ‘not hard’; no chronic burden due to ongoing difficulties with job
higher			
			
20	153	41.8	Physical activity in the past year is moderately active or less than moderately active, or physically inactive; chronic burden due to serious on-going personal health problem (yes, but not very stressful or yes, moderately stressful or yes, very stressful); can trust neighbours; grade of school completed is higher than college
lower			
			
27	53	41.5	Physical activity in the past year is moderately active or less than moderately active, or physically inactive; no chronic burden due to serious ongoing personal health problem; grade of school completed is higher than college; history of high blood pressure; cigarettes smoked per day is <= 12.5
lower			
			
28	58	37.9	Physical activity in the past year is moderately active or less than moderately active, or physically inactive; no chronic burden due to serious ongoing personal health problem; grade of school completed is higher than college; history of high blood pressure; cigarettes smoked per day is >= 12.5
lower			
			
24	191	37.2	Physical activity in the past year is moderately active or less than moderately active, or physically inactive; no chronic burden due to serious ongoing personal health problem; grade of school completed is less than college; degree of difficulty paying for basics is ‘not hard’; chronic burden due to ongoing difficulties with job (yes, but not very stressful, or yes, moderately stressful, or yes, very stressful)
lower			
			
16	251	31.5	Physical activity in the past year is moderately active or less than moderately active, or physically inactive; no chronic burden due to serious ongoing personal health problem; grade of school completed is less than college; degree of difficulty paying for basics is ‘hard’
lower			
			
19	158	22.8	Physical activity in the past year is moderately active or less than moderately active, or physically inactive; chronic burden due to serious on-going personal health problem (yes, but not very stressful or yes, moderately stressful or yes, very stressful); can trust neighbors; grade of school completed is less than college
lower			
			
12	281	15.3	Physical activity in the past year is moderately active or less than moderately active, or physically inactive; chronic burden due to serious on-going personal health problem (yes, but not very stressful or yes, moderately stressful or yes, very stressful); cannot trust neighbors
lower			
	**Total 3648**		

The characteristics of individuals in three subgroups (terminal nodes labelled 22 and 12, and terminal node 23) in the tree model were each compared with the rest of the study sample based on psychosocial and socioeconomic variables that did not appear as splitting variables in the final tree model:

(1) Subgroup (node) 22 (bottom left of Fig. 1) had the largest proportion of higher SRH (92.9%, n=560). Node membership was characterized by higher physical activity rating (more than moderately active, or very active); higher income category (>$25,000-$34,999); no chronic burden due to personal serious on-going health problem (or if present, not very stressful); no history of hypertension; highest year of school completed is graduate level.
(2) Subgroup (node) 12 (middle right of Fig. 1) had the largest proportion of lower SRH (84.7%, n=238). Node membership was characterized by lower physical activity rating; chronic burden due to personal serious on-going personal health problem; and perception that people in the neighbourhood could not be trusted.

Comparing proportions with z tests, membership in subgroup 22 (largest proportion higher SRH) was also associated with being white; owning a home; being employed; feeling that friends and family care, and can be relied upon; perception of neighbours helping each other/getting along/sharing values; and the neighbourhood being close knit. Subgroup 22 had a significantly larger proportion of respondents who felt that they had control over life events, were not helpless dealing with life problems, and were optimistic for the future.

Membership in subgroup 12 (largest proportion lower SRH) was associated with being black, not owning a home, being unemployed, not feeling that family and friends care, or can be relied upon for support, perception that neighbours don’t help each other, the neighbourhood is not close knit, that neighbours don’t get along, and don’t share values. A larger proportion of respondents felt they had no control over life events, felt helpless dealing with life problems, and were not optimistic for the future.

Terminal node 23 had predominantly higher SRH status (but the lowest overall proportion at 54.9%). Membership in node 23 was associated with physical activity rating of moderately active, less than moderately active, or physically inactive; education less than college-level; no chronic burden due to serious ongoing personal health problem; no chronic burden due to ongoing difficulties with job; degree of difficulty paying for basics perceived as ‘not hard’. Comparing individuals in this node with the rest of the study sample, membership was also associated with being black, perception that neighbours could not be trusted, and feeling helpless dealing with life problems.

### Random forests analysis

In the random forests analysis, physical activity, income and education, were the highest ranking variables associated with SRH. These variables had the greatest decreases in node impurity, (137.419, 112.478, 88.903, respectively), and reflected the highest variable importance ranking (Table 2). Age was ranked fourth (78.727) and chronic burden due to a serious personal health problem was ranked fifth (77.353). Most of the predictor variables indicating history of a specific medical condition were ranked relatively low, apart from high blood pressure (37.232) and high cholesterol (17.643). The out-of-bag error rates for the random forests model varied depending on the number of trees specified for the model. There was no major decrease in error above approximately 300 trees. The overall estimated out-of-bag error rate was 26%, compared with the cross-validated error estimate of 31% for the single classification tree.

**Table 2:** Random forests variable importance ranking, the CARDIA Study, Year 15, USA (variables with value for decrease in node impurity >15).

**Variable Importance**	
	**Decrease in Node Impurity**^a^
Physical activity	**137.419**
Income	**112.478**
Education	**88.903**
Age	**78.727**
Chronic burden – personal health problem	**77.353**
Fast food consumption	**66.217**
Chronic burden – financial strain	**57.509**
Beer consumption	**43.349**
Chronic burden serious health problem – other person	**43.275**
Chronic burden – job/work	**43.195**
Chronic burden - relationship	**43.149**
High blood pressure	**37.232**
Wine consumption	**36.257**
Cigarettes/day	**35.517**
Liquor consumption	**31.220**
Difficulty paying for basics	**28.406**
Optimism for future	**27.367**
Race/ethnicity	**27.261**
Neighborhood trust	**23.361**
Neighbors share values	**20.094**
Neighbors help each other	**19.213**
Control over life events	**18.070**
High cholesterol	**17.643**
Sex	**17.206**
Maternal high blood pressure	**16.416**
Neighbors get along	**16.123**
Close-knit neighborhood	**16.023**
Marijuana use	**15.458**
Paternal high blood pressure	**15.404**

a Total decrease in node impurities from splitting on the variable averaged over all trees (by Gini index).

## Discussion

Findings from this study suggest that, in the CARDIA sample, a range of multi-domain factors are associated with SRH. CTA indicates that profiles of risk factors associated with SRH are not uniform between different subgroups, including those with similar health status. Comparison of the subgroups with the largest and smallest proportions of higher SRH (node 22: 92.9% and node 12; 15.3%, respectively) revealed combinations of factors from multiple domains of health as potentially relevant to SRH status including race/ethnicity, physical activity level, income and education, chronic burden due to on-going personal health problem, neighbourhood factors, perception of control over life events, and optimism for the future. The single classification tree reflected interaction of lifestyle and medical factors with income and education; for individuals with similar levels of physical activity or chronic burden related to a serious personal health problem, subgroups with higher income or education were also those with higher proportions of higher SRH. Chronic burden due to serious ongoing personal health problem ranked highly in terms of relative importance associated with SRH. Despite the inclusion of many specific medical conditions, and variables regarding access to services and medical insurance, these ranked low, apart from high blood pressure. This finding is of interest as in earlier studies on the CARDIA study cohort, high blood pressure has been shown to be associated with subclinical outcomes such as coronary artery calcification and carotid intima-media thickening; high blood pressure in early adulthood has also been noted as an important antecedent of heart failure; thus this appears to be an important medical risk factor to target for early prevention (Bibbins-Domingo et al., 2009, Loria et al., 2007, Polak et al., 2010). Though the predictor variable profiles for the 15 subgroups are varied, for the whole study sample, by random forests analysis, physical activity and socioeconomic variables, education and income, were highly ranked in association with SRH status.

CTA and parametric models may highlight some similar individual covariates of SRH – indeed in this study predictor variables are included as they represent known determinants of health. However the production of 15 subgroups in the tree model indicates that the same set of factors do not affect SRH in the same way across the whole sample; this is reflected in Table 1 which lists summary descriptions of some of the key factors associated with SRH in the 15 subgroups, and in the more detailed results of nodes 22, 12, and 23 Some factors are associated with subgroups that are predominantly higher SRH, and with subgroups that are predominantly lower SRH (for example physical activity level of moderately active/less than moderately active/physically inactive; highest grade of education as college; ability to trust neighbours). More detailed comparison of subgroups 22 and 12 show a clustering of protective factors associated with higher SRH, and a clustering of negative risk factors associated with lower SRH. Interaction of behavioural factors and income and education is also apparent.

CTA reveals variability in outcome, depending on varying combinations of risk and protective factors; this has relevance for actions to improve SRH. Public health interventions may need to address multiple factors, from different domains, and consider their interactions and relative importance in prioritizing action to improve health status. Social contextual factors (education, income, personal resources) are important in influencing health behaviors like physical activity (Emmons, 2000). For interventions to be effective, acknowledging the socioeconomic context of health behaviors and other risk factors is vital when designing and implementing health promotion and disease prevention strategies, particularly in the context of limited resources (Winkleby, Cubbin, Ahn, & Kraemer, 1999).

Outcomes such as SRH status may be the result of a broad array of factors that interact and impact upon individuals in different ways. The extent to which this occurs, and results in differential risk or protective factors for different subgroups may be unknown, and therefore identifying the most relevant risk or protective factors, on which to focus intervention efforts may be challenging (BeLue, Francis, Rollins, & Colaco, 2009). Recursive partitioning is useful in describing such associations, patterns and structure in data (Friel et al., 2005, Lemon et al., 2003). Parametric regression models are essential in the testing of hypotheses of the impact of single independent variable, or small sets of variables, on an outcome measure; they are less suited to the analysis of high dimensional datasets with various classes of data, as used in this study, and in demonstrating the full interplay of factors relating to SRH. Although there are methods to handle missing data in the context of logistic regression, the standard is complete case analysis, and so an observation will be dropped if any covariate or outcome data is missing. In the case of this study, complete case analysis would drop effective sample size from 3648 to 258 due to exclusion of individuals with missing data. Conversely, if the tree model does not split on a variable, then its missingness does not diminish the data used for making the tree. The analytic approach used in this study is not dependent on the data following a particular distribution. This is pertinent given the aim of simultaneously considering categorical, ordinal, and continuous variables from several health-related domains. Classification trees are a non-parametric, data-adaptive method and so do not assume an *a priori* mod*el.* Thus, they are better suited than pre-specified regression models for finding unspecified predictive combinations of variables and thus, susceptible subgroups. The tree-based variable importance measure captures both linear and arbitrarily non-linear joint relationships among covariates and the outcome, whereas logit-linear only captures joint linear relationships; so relative importance is only interpretable if the true model is logit-linear. Breiman describes statistical modelling as having two cultures: (1) data modelling assumes a stochastic data model; (2) algorithmic modelling treats the data mechanism as unknown. In the first approach, with complex high dimensionality datasets, including different types of variables, there is a risk of making incorrect assumptions on the structure of the underlying data being multivariate normal. Breiman argues, *“If the model is a poor emulation of nature, the conclusions may be wrong”* (Breiman, 2001b). In high dimensional datasets, traditional regression approaches can also produce model parameters with little real world interpretability (Sudat, Carlton, Seto, Spear, & Hubbard, 2010).

There are limitations to this study. Interpretation of the tree is exploratory, and results are considered in that manner. Though the predictor variables were selected from an existing strong dataset to represent multiple layers of influences on health, a few may not optimally represent the characteristic of interest. For example, diet is included only by way of fast food intake. Responses that were classed as ‘don’t know’ in the original CARDIA data collection were labelled in this study as ‘missing’. It is possible that this could introduce some bias if people who responded to certain questions with ‘don’t know’ were more likely to have a particular self-rated health status. Dichotimising the outcome (and other variables) results in a degree of loss of information; as an exploratory study, this was balanced against the potential of a very complex tree model, e.g. if five SRH categories were preserved. Some variables were dichotomized prior to the analysis. The tree model can also create a cut-off point and in effect artificially dichotomize the variable based on the splitting of the dataset at that node. However, not all splits are based on dichotomization as some are based on existing groupings e.g. income categories, and others on continuous variables. The form of the outcome (dichotomous versus continuous for instance) is not relevant to the use of trees over parametric regressions (both can handle different outcome types).

Tree-based methods are prone to instability, so that small perturbations in the data can produce large variations in tree structure even though prediction accuracy might not vary at all, though this may be less problematic since the focus here is on understanding specific influences within one population group. The application of random forests attempts to address this; the ensemble of trees improves predictive accuracy and provides more robust variable importance measures. Even so, in using a non-probabilistic method, there is no rigorous theory for providing inference on the structure of the tree, and the output of random forests too, in the absence of a Type 1 error rate, is best considered a rank ordering of key variables worthy of further investigation. An additional issue is that though recursive partitioning methods are efficient at uncovering interactions but compared to standard regression models, may miss variables which have relatively weak but uniform effects across all individuals in the sample (Ciampi, Thiffault, Nakache, & Asselain, 1986). The overall misclassification rate for the single tree of 31% is similar to that found in previous studies using CTA (BeLue et al., 2009, Friel et al., 2005). Though similar data were collected in later years, the focus here was on a young adult population. As an exploratory study, we do not suggest that these results are widely generalizable but seek to demonstrate the usefulness of this approach in understanding specific needs and risk factor profiles which affect health outcomes in different populations and subgroups.

Newer recursive partitioning techniques do address some of these limitations. Bagging trees consists of multiple trees grown out of bootstrap samples that are combined by averaging (for regression) or by simple vote for classification (Sutton, 2005). Random forests add the further dimension of a random sampling of the predictor variables. Random predictor selection controls the bias (Prasad, Iverson, & Liaw, 2006). Random forests are a valuable tool, particularly when used in conjunction with classification tree analysis, producing more robust measures of variable importance. There is some loss of interpretability with random forests, as trees are not graphically represented due to large numbers, and though it is a very good predictor, there are limitations in interpretation due to an inherent over-fitting. As a result, it may systematically underestimate the importance of variables, given a phenomenon equivalent to over-adjustment in more standard epidemiological parlance (Schisterman, Cole, & Platt, 2009).

Other semi-parametric methods can build on this type of analysis. Using a counterfactual approach to generate variable importance, the distribution of the outcome of interest can be compared with its theoretical distribution if the variable of interest is set to the lowest risk (Hubbard and Laan, 2008, Sudat et al., 2010). This is especially useful in producing parameters that translate well to public health practice. Variable importance analysis by fitting multiple Population Intervention Models (PIMs) produces a parameter that is analogous to attributable risk. Under certain assumptions, the parameter can be considered as an actual causal effect of the exposure variable on the outcome, or as measuring the hypothetical effect of an intervention in which everyone in the population is made to be like the members of the target group (Hubbard and Laan, 2008, Ritter et al., 2011). The advantage of these methods is that they can use the power of techniques such as random forests that are very good at flexibly fitting the data, while still providing interpretable and robust estimates of variable importance.

Alternative non-parametric approaches to those applied in this study exist, but these have different drawbacks. Dimension reduction with principal components or factor analysis, results in the original predictor variables being transformed into a reduced set of components. However, their individual effect is no longer clearly identifiable (Strobl et al., 2009). Portrait et al. recognized single elements of the broad range of health determinants reflect only some aspect of health but without consideration of cofactors, are incomplete predictors of overall health status, and discussed the difficulties of processing the rich set of indicators needed to capture the concept of health. They applied Grade of Membership analysis to form a typology of elderly individuals’ health status and conceptualized health status or outcome as graded participation into several aspects of health (Portrait et al., 1999). Results are generated as a number of hypothetical pure types or groups, along with numerical weightings of the affinity of individuals with pure types. Though this approach recognizes the multidimensionality of health data, for some research questions, this type of output is not easily translatable to be of value in practice.

The social-ecological model offers a theoretical framework for understanding the dynamic interplay among persons, groups, and their socio-physical environments; health promotion efforts based on this model need to be informed by knowledge of the links between numerous aspects of health status and the joint influence of multi-domain factors (Stokols, 1996). Though focused on individual-level data, recursive methods in this study reflect this perspective; the results capture multiple influences on SRH, and reflect their interactions. In addition, they identify subgroups of the sample with common profiles of characteristics, and indicate relative importance of factors in relation to health status. The latter is useful particularly as one of the criticisms leveled at the social-ecological model is that they are too comprehensive in nature (Green, Richard, & Potvin, 1996); random forests produce an important ranking of variables, which suggest where action could be prioritized to improve SRH status, addressing both individual and upstream factors.

The application of recursive partitioning methods to study correlates of health is analogous to an audience segmentation approach (Lemon et al., 2003). Classification tree methods add a valuable dimension by not only grouping based on like factors, but also modeling multiple factors of interest on an outcome; health promotion activities could subsequently be tailored to specific needs in groups. Audience segmentation originated in commercial marketing, seeking to understand the customer. It has been adopted in public health to gain knowledge of communities and population groups, and to inform social marketing, a method of achieving behavior change with lifestyle modification through targeted health promotion programs (Choosing Health: Making healthy choices easier. Public Health White Paper, 2004). Segmentation may also generate information that can influence policy makers who can address the relevant social and environmental determinants of health found to be of most importance in population groups (Grier & Bryant, 2005). These ‘causes of the causes’ may be more difficult to remedy but are important in relation to enabling good health and, as the classification tree suggests in this study, may interact with individual level factors, influencing individual behavior (Marmot, 2007).

Recursive partitioning analysis may be a better reflection of how multiple influences on health interact in reality. Kaplan et al. highlighted the importance of a public health approach that, “does not exclusively privilege the proximal, [or focus on molecular explanations of disease] but seeks opportunities for understanding and intervention at both upstream and downstream vantage points” (Kaplan, Everson, & Lynch, 2000).

## Conclusion

This study demonstrates the utility of recursive partitioning in extending segmentation principles to explore combinations of multi-domain risk and protective factors related to health outcomes. SRH status is an independent predictor of future health –related outcomes. Therefore identifying factors linked to higher/lower status at younger ages suggests where action could be prioritized and targeted to better current SRH status, and subsequently improve future health-related outcomes. Understanding the drivers of illness and wellbeing, and their relative importance in specific groups provides a basis for developing and prioritizing targeted action for those individuals, with interventions that are most appropriate to need.

## Figures and Tables

**Table A1:** Predictor variables used in classification tree analysis CARDIA Study, Year 15, USA.

**Variable**	**Description**
**Age, sex and hereditary factors**	
**Age**	Age in years
**Sex**	Male or Female
**Race/ethnicity**	Hispanic, black (not Hispanic), white (not Hispanic) a
**Family History**	History of maternal or paternal diabetes, high blood pressure, stroke, angina, heart attack

**Individual lifestyle factors and medical history**	
	
**Medical history – presence of disease**	History of disease for each condition:
	high blood pressure; high blood cholesterol; heart disease; asthma; chronic bronchitis; emphysema; diabetes; liver disease; kidney disease (excluding nephritis or glomerulonephritis); cancer or malignant tumour; HIV; stroke or TIA (transient ischemic attack); multiple sclerosis; epilepsy (seizures); nervous / emotional or mental disorder; depression
**Diet**	Number of times per week that breakfast, lunch, or dinner eaten out in fast food restaurant such as McDonald’s, Burger King, Wendy’s, Arby’s, Pizza Hut, or Kentucky Fried Chicken
**Physical activity**	5 point rating of physical activity compared to other people of same age and sex during the past year? (1=physically inactive to 5=very active)
	
**Smoking / Tobacco**	History, for at least 3 months, of being regular cigarette smoker (at least 5 per week almost every week)
	Still smoke cigarettes regularly (at least 5 per week almost every week)
	Number of cigarettes smoked per day on average (1 pack=20 cigarettes) (continuous variable)
	
**Alcohol**	Number of drinks per week of wine (about a 5 oz. glass).
	Number of drinks per week of beer (1 beer is a 12 oz. glass, can, or bottle).
	Number of drinks per week of hard liquor (each shot of 1½oz. counted as 1 drink).
	
**Illicit drug use**	History of drug use for ever using:
	marijuana / crack / other forms of cocaine that are not crack (including powder, free base, and coca paste) / amphetamines (“Speed” or “Uppers”) / opiates for non-medical reasons (Heroin, Dilaudid, Morphine, Demerol)?

**Social and community influences**	
	
**Social support / network (“feeling that family friends really care”)**	Family members or friends are perceived to care
	Can rely on family members or friends if need to talk about worries.
	
**Sense of close knit neighborhood, neighborhood cohesion**	In thinking about the neighborhood in which you live:
	People willing to help their neighbors / Live in close-knit neighborhood.
	People in the neighborhood can be trusted.
	People in the neighborhood generally get along with each other.
	People in the neighborhood share the same values

**Living and working conditions**	
	
**Education**	Highest grade (or year) of regular school completed?
	01–08=elementary school
	09–12=High School
	13–16=College
	17–20+=Graduate School
	
**Income**	Total combined family income for the past 12 months?
	1 = Less than $5,000
	2 = $5,000 - $11,999
	3 = $12,000 - $15,999
	4 = $16,000 - $24,999
	5 = $25,000 - $34,999
	6 = $35,000 - $49,999
	7 = $50,000 - $74,999
	10=$75,000 - $99,999
	11=$100,000 and greater
**Housing - rent or own house**	Own home versus rented, occupied or other
**Employment - working versus unemployed**	Unemployed status
	
**Control & adequacy of resources (“how hard is it to pay for basics”)**	Hard to pay for basics
	Hard to pay for medical care
	
**Medical insurance**	Always had health insurance or other coverage for medical care in the past two years.
	Covered by health insurance like Blue Cross/Blue Shield or participation in an HMO; health insurance obtained through an employer, union, or school.
	Self-insured
	
**Access to health services**	Categorical indicator variables:
	Did not seek medical care in past 2 years due to cost
	Has been hard overall getting health services
	1=’hard’ (very /fairly)
	0=’not hard’ (not too hard/not hard at all)
**Experience of discrimination due to:**	Experience of discrimination due to gender / race-ethnicity or color / socioeconomic position or social class for each setting:
	
**Gender**	At school Getting a job Getting housing At work At home Getting medical care On the street or in a public setting
**Race/ethnicity or colour**	
**Socioeconomic position or social class**	
	
**Some type of on-going chronic burden**	Experienced strains for longer than 6 months due to
	Serious on-going health problem (yourself).
	Serious on-going health problem (someone close to you).
	On-going difficulties with your job or ability to work
	On-going financial strain
	On-going difficulties in a relationship with someone close to you
	1=No
	2=Yes, but not very stressful
	3=Yes, moderately stressful
	4=Yes, very stressful
	
**Optimism for the future**	Have no control over the things that happen
	Feel helpless in dealing with the problems of life
	Always optimistic about future

a CARDIA was designed to be a biracial cohort, however, information on ethnicity was collected, and this is reflected by the 11 participants classified in the study as Hispanic.

**Table A2:** Relationship between selected predictor variables and SRH in the CARDIA Study, Year 15, USA.

**Predictor variables**		**SRH category**				
		**‘Lower’ n=1514**		**‘Higher’ n=2134**		**Chi-square for independence**
		**Count (column %)**	**%**	**Count (column %)**	**%**	
**Sex**	Male	626 (41.3)	38.8%	986 (46.2)	61.2%	p=0.004*
	Female	888 (58.7)	43.6%	1148 (53.8)	56.4%	
						
**Race/ethnicity**	Hispanic	5 (0.33)	45.5%	6 (0.28)	54.5%	P=0.000*
	Black	887 (58.6)	51.7%	830 (38.9)	48.3%	
	White	622 (41.1)	32.4%	1298 (60.8)	67.6%	
						
**Physical activity rating**	1	170 (11.2)	72.6%	64 (3.0)	27.4%	P=0.000
	2	372 (24.6)	60.2%	246 (11.5)	39.8%	
				
	3	730 (48.2)	45.3%	881 (41.3)	54.7%	
**1= physically inactive**						
**5=very active**						
	4	142 (9.4)	22.5%	490 (23.0)	77.5%	
	5	96 (6.3)	17.6%	448 (21.0)	82.4%	
						
**Still smoke cigarettes regularly**^a^	No	264 (17.4)	38.9%	415 (19.4)	61.1%	P=0.000*
	Yes	452 (29.9)	56.4%	350 (16.4)	43.6%	
**Social support**	No	56 (3.7)	62.9%	33 (1.5)	37.1%	P=0.000*
**Family members/friends perceived to care**	Yes	1457 (96.2)	40.9%	2102 (98.5)	59.1%	
						
**Live in close-knit neighborhood**	No	971 (64.1)	46.1%	1137 (53.3)	53.9%	P=0.000*
	Yes	543 (35.9)	35.3%	997 (46.7)	64.7%	
						
**Total family income**	Less than $5,000	67 (4.4)	77.0%	20 (0.9)	23.0%	P=0.000
	$5,000 - $11,999	97 (6.4)	69.3%	43 (2.0)	30.7%	
	$12,000 - $15,999	66 (4.4)	58.9%	46 (2.2)	41.1%	
	$16,000 - $24,999	131 (8.7)	54.8%	108 (5.1)	45.2%	
	$25,000 - $34,999	183 (12.1)	53.5%	159 (7.4)	46.5%	
	$35,000 - $49,999	268 (17.7)	47.1%	301 (14.1)	52.9%	
	$50,000 - $74,999	305 (20.1)	38.6%	486 (22.8)	61.4%	
	$75,000 - $99,999	193 (12.7)	36.6%	334 (15.6)	63.4%	
	≥ $100,000	183 (12.1)	23.0%	614 (28.8)	77.0%	
						
**Own home**	No	582 (38.4)	51.2%	554 (25.9)	48.8%	P=0.000*
	Yes	929 (61.4)	37.1%	1577 (73.9)	62.9%	
						
**Unemployed**	No	1324 (87.5)	40.1%	1980 (92.7)	59.9%	P=0.000*
	Yes	185 (12.2)	55.6%	148 (6.9)	44.4%	
						
**Had health insurance past 2 years**	No	241 (16.0)	52.4%	219 (10.3)	47.6%	P=0.000*
	Yes	1269 (83.8)	39.9%	1912 (90.0)	60.1%	
						
**Difficulty paying for basics**	No	1054 (69.6)	36.2%	1860 (87.1)	63.8%	P=0.000*
	Yes	452 (29.9)	62.6%	270 (12.6)	37.4%	
						
**Optimistic for future**	No	578 (38.1)	54.5%	482 (22.6)	45.5%	P=0.000*
	Yes	936 (61.8)	36.2%	1652 (77.4)	63.8%	
						
**Control over life events**	No	341 (22.5)	60.1%	226 (10.6)	39.9%	P=0.000*
	Yes	1173 (77.5)	38.1%	1907 (89.3)	61.9%	
						
**Serious personal ongoing health problems (self) > 6 months**	1 no	1003 (66.2)	34.6%	1898 (88.9)	65.4%	P=0.000
	2 yes, not very stressful	174 (11.5)	60.2%	115 (5.4)	39.8%	
	3 yes, moderately stressful	200 (13.2)	73.5%	72 (3.4)	26.5%	
	4 yes, very stressful	137 (9.0)	73.7%	49 (2.3)	26.3%	
	**Total**	**1514**	**41.5%**	**2134**	**58.5%**	

* Continuity correction

## Appendix A

See Appendix Table A1, Table A2.
