# Supplementary material for: A recursive partitioning approach to investigating correlates of self-rated health: The CARDIA Study
Source: SSM Popul Health. 2017 Dec 15;4:178–88. doi: 10.1016/j.ssmph.2017.12.002 (PMC5976867; doi:10.1016/j.ssmph.2017.12.002)
Supplement: Supplementary file 1 — Supplementary material [file mmc1.doc]

**Supplementary information**

The work was carried out as part of my doctoral research at the University of California, Berkeley, USA, (published as a dissertation). This manuscript is not under consideration for publication in any other journal. There is one related manuscript (submitted) from my doctoral research, which uses classification tree analysis on the same CARDIA data sample but to contextualize factors by socioeconomic group (a portion of that manuscript was presented at the 2014 Society for Social Medicine Annual Scientific Meeting-poster presentation published as an abstract).

Author contributions are as follows:

SN conceived the study and its design, conducted data analyses and wrote the first draft of this manuscript.

AH contributed to study design and analyses.

SS contributed to data analyses.

SLS contributed to the conception of the study, and its design.

All authors contributed to interpretation of data, and revision of the manuscript. All authors have approved the final version of this manuscript.

All authors have no competing interests. All authors have no financial disclosures.

Thank you for considering this manuscript for publication in *Social Science & Medicine – Population Health.* We look forward to your response.

Yours sincerely,

Shilpa Nayak

Shilpa Nayak MBChB(Hons), MPH, MFPH, PhD

Department of Public Health and Policy, The Whelan Building, Quadrangle,

The University of Liverpool L69 3GB, UK

Email: shilpan@liv.ac.uk
